# Unraveling the Role of the Apical Papilla During Dental Root Maturation

**DOI:** 10.3389/fcell.2021.665600

**Published:** 2021-05-06

**Authors:** Ronald B. Driesen, Pascal Gervois, Tim Vangansewinkel, Ivo Lambrichts

**Affiliations:** Faculty of Medicine, Hasselt University, Biomedical Research Institute, Cardio and Organ Systems, Hasselt, Belgium

**Keywords:** apical papilla, SCAP, dental, root, development

## Abstract

The apical papilla is a stem cell rich tissue located at the base of the developing dental root and is responsible for the progressive elongation and maturation of the root. The multipotent stem cells of the apical papilla (SCAP) are extensively studied in cell culture since they demonstrate a high capacity for osteogenic, adipogenic, and chondrogenic differentiation and are thus an attractive stem cell source for stem cell-based therapies. Currently, only few studies are dedicated to determining the role of the apical papilla in dental root development. In this review, we will focus on the architecture of the apical papilla and describe the specific SCAP signaling pathways involved in root maturation. Furthermore, we will explore the heterogeneity of the SCAP phenotype within the tissue and determine their micro-environmental interaction. Understanding the mechanism of postnatal dental root growth could further aid in developing novel strategies in dental root regeneration.

## Introduction

**FIGURE 1:**
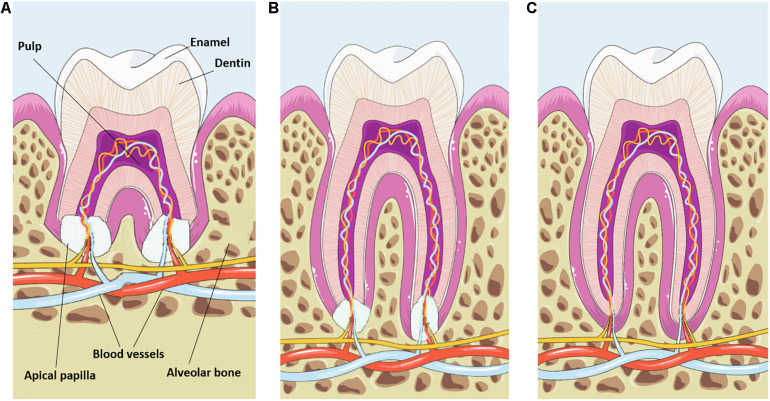
Schematic representation of apical papilla-induced root maturation. **(A)** The apical papilla is located at the growing part of the maturing dental root. Note the different components of the tooth and the dental vasculature entering the apical papilla and pulp tissue. **(B)** During root elongation the apical papilla progressively decreases in size. **(C)** Fully mature roots are associated with a complete loss of the apical papilla tissue. This image was created using Servier Medical Art, licensed under a Creative Common Attribution 3.0 Generic License, available online at https://smart.servier.com/.

Dental tooth development can be subdivided into different steps and starts with the formation of the crown during the bud, cap and bell stages. Once the crown has taken shape, the dental root starts to grow under coordination of the Hertwig’s epithelial root sheath (HERS) (Luan et al., 2006). This double layer of the epithelial sheath grows apically and positions itself between the dental papilla and dental follicle. During root elongation and formation of dentin, the HERS will be fragmented into the epithelial rests of Malassez allowing dental follicle cells to establish contact with the newly formed dentin and to differentiate into cementoblasts (Huang X. et al., 2009). Collagen fibers secreted by dental follicle cells are deposited against the cementum to enable a firm connection to the alveolar bone (Zhou et al., 2019). Consequently, tooth root formation and elongation is associated with eruption and positioning of the newly formed tooth. Complete elongation and maturation of the dental root is guided by the apical papilla (Figure 1) which originates from the ectomesenchyme (Huang et al., 2008). From a developmental point of view, the architectural composition of the apical papilla and its role in dental root maturation are poorly studied. Here, we will provide an in depth overview on the current knowledge of the apical papilla tissue and how the stem cell rich content drives dental root development through specific signaling pathways and micro-environmental changes.

From a macroscopic point of view, the apical papilla is located apical to the epithelial diaphragm and is partitioned from the dental pulp by a cell-rich zone (Sonoyama et al., 2008). Within the collagenous-rich matrix of the apical papilla, a high concentration of mesenchymal stem cells reside which are defined as stem cells of the apical papilla (SCAP). These highly proliferative SCAP will contribute to the formation of dentin by differentiating into odontoblasts and/or are recruited to the connected pulp tissue. Since the discovery of the SCAP within the apical papilla, it became clear that multipotent SCAP are highly suitable for osteogenic, adipogenic and chondrogenic differentiation (Huang G. T. J. et al., 2009). Together with their immunomodulatory capacity (Gaudin et al., 2018; Liu et al., 2019; Fehrmann et al., 2020), SCAP have a high potential for implementation in tissue regeneration. This offered the research community an easily accessible mesenchymal stem cell source with low ethical considerations. Recently, our group identified novel histological regions (Driesen et al., 2020) based on the collagen distribution and organization. At the surface layer, the connective tissue is organized as a dense collagen matrix with perpendicular aligned collagen fibers and which is defined as cortex fibrosa. The inner part of the apical papilla which is indicated as medulla consists of a loosely disorganized collagen matrix with a high concentration of SCAP. The whole apical papilla is furthermore encapsulated by a single layered cuboid epithelium and can be considered as an independent tissue structure with its own vascular network and innervation branching toward the central dental pulp vasculature and nerve. Therefore, the apical papilla is less susceptible to pathologic events leading to pulp necrosis and to apical periodontitis (Chepra et al., 2017). In addition SCAP have been demonstrated to own a high immune stability profile (Lei et al., 2021). Furthermore, resection of the dental pulp via pulpectomy (Jung et al., 2011) or periapical lesion after trauma (Jiang and Liu, 2020) with preservation of the apical papilla revealed that root maturation is continuous and is independent from the dental pulp.

## Molecular Mechanism of Root Formation

**FIGURE 2:**
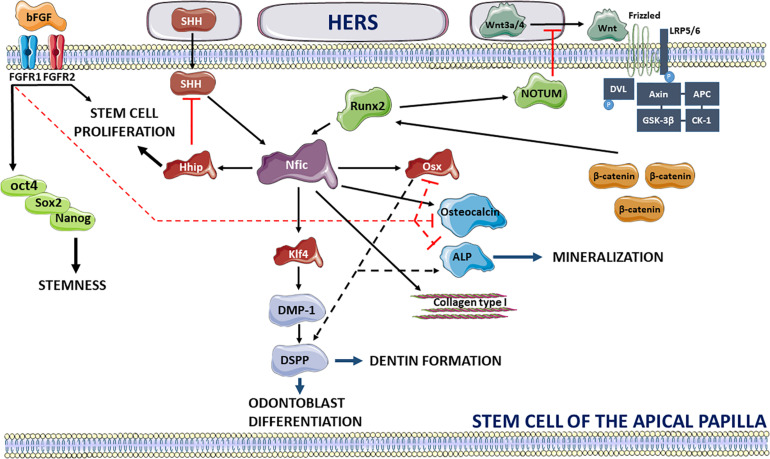
Overview of signaling pathways regulating the stemness of SCAP and promoting dental root development via extrinsic signals of HERS. bFGF maintains the stemness of SCAP via activation of FGFR1 and 2 promoting expression of stemness factors oct4, Sox2, and Nanog, inhibits mineralization through interaction with Osx, Osteocalcin, and ALP and promotes cell proliferation. HERS stimulates dental root maturation by secretion of Wnt3a and Wnt4 interacting with the Frizzled and LRP5/6 receptors. Activation of Wnt signaling acts via binding and phosphorylation of DVL, phosphorylation of LRP5/6 and de-activation of the Axin, APC, GSK-3β, and CK-1 complex leading to inhibition of β-catenin degradation. Consequently, β-catenin interacts with Runx2 activating the Nfic pathway. Nfic triggers odontoblast differentiation and dentin formation by acting with Klf4 and promoting DMP-1 and DSPP expression. Secondly, Nfic induces mineralization by upregulating Osteocalcin, ALP and collagen type I and through interaction with Osx which promotes ALP production and DSPP expression. Alternatively, HERS activates Nfic signaling independent of Runx2 via secretion of SHH. Wnt and hedgehog signaling pathways are, respectively, antagonized by Runx2-driven secretion of NOTUM and Nfic-promoted expression of Hhip which promotes stem cell proliferation. Activators of signaling pathways are shown in black lines (full and dotted) whereas inhibition is depicted in red. This image was created using Servier Medical Art, licensed under a Creative Common Attribution 3.0 Generic License, available online at https://smart.servier.com/.

Recent studies have procured more information on the signaling pathways specifically involved in tooth root development. An important prerequisite for the start of dental root growth after crown formation is the de-activation of fibroblast growth factor-10 signaling in the dental papilla (Yokohama-Tamaki et al., 2006). At the apical site of root development, a dynamic interaction of SCAP in close vicinity with the HERS is observed which influences apical root morphogenesis and controls root number, length and dentin formation (Luder, 2015; Figure 2). HERS stimulates root formation in the mesenchyme of the apical papilla through secretion of Wnt3a and 4 and sonic Hedgehog (SHH) which upregulate the expression of the Nfic transcription factor, the central regulator for root formation. HERS triggers the Nfic pathway in SCAP by switching on the canonical Wnt pathway which has been shown to be essential for postnatal root maturation (Kim et al., 2013; Wang et al., 2020). HERS-secreted Wnt3a and Wnt4 proteins will couple to the seven-pass transmembrane Frizzled receptor and the low-density lipoprotein receptor-related protein 5 or 6 (LRP5/6) (Wen et al., 2020). Recruitment of Disheveled (DVL) promotes phosphorylation of the LRP6 receptor and binding of the Axin complex composed of tumor suppressor adenomatous polyposis coli gene product (APC), glycogen synthase kinase 3 (GSK3) and casein kinase 1 (CK1) (MacDonald et al., 2009). This will lead to a reduction of β-catenin phosphorylation and degradation allowing β-catenin to upregulate the expression of runt-related transcription factor 2 (Runx2) followed by direct activation of the Nfic pathway. The importance of β-catenin in root development has previously been shown in conditional knockout mice for β-catenin revealing impaired root elongation (Yang et al., 2020) and loss of HERS structural integrity. Consequently, activation of the Nfic signaling pathway will promote odontoblast differentiation and dentin formation via Krüppel-like factor 4 (Klf4) expression resulting in the production of dentin matrix acidic phosphoprotein-1 (DMP-1) and dentin sialophosphoprotein (DSPP) (Lee et al., 2014). Nfic-induced mineralization is stimulated via increased expression of collagen type I and osteocalcin (Zhang et al., 2015) and through upregulation of osterix (Osx) which activates ALP production and induces DSPP expression (He et al., 2016). Loss of Osx expression has been shown to result in failure of complete root maturation and a decrease in odontogenic differentiation (Zhang et al., 2015). The interplay between Runx2 and Wnt signaling, however, is considered complex and requires a delicate regulation for achieving optimal root elongation (Wen et al., 2020). Overexpression of Wnt signaling results in shorter roots whereas de-activation completely arrests root development. This balance is guided by the Runx2-induced secretion of NOTUM, an inhibitor of the Wnt signaling pathway by de-acetylating Wnt3a (Wen et al., 2020). It should be noted that the Ror2 mediated non-canonical Wnt signaling and its downstream Cdc42 mediator has revealed an impact on the mesenchyme cell proliferation and a contribution to root development size in mouse molars (Ma et al., 2021). Secondly, HERS triggers tooth root development by secretion of SHH which is regulated in the epithelial compartment by SMAD4-mediated transforming growth factor-beta (TGF-β)/bone morphogenetic protein (BMP) signaling (Huang and Chai, 2012). SHH contributes to the activation of the Nfic signaling pathway independent of Runx2 resulting in elevated expression levels of hedgehog interacting protein (Hhip) and promotion of SCAP proliferation (Liu Y. et al., 2015). It has been shown that hedgehog signaling activity is the highest in SCAP at the periphery of the apical papilla and diminishes toward the center of the tissue creating a concentration gradient (Li et al., 2015). In addition, the level of hedgehog activity is negatively regulated by the Nfic-activated Hhip ensuring proper root formation (Liu Y. et al., 2015).

Once root growth is initiated, apical odontoblasts emerge at the base of the developing roots displaying the expression of the aforementioned transcription factors i.e., Nfic, Osx, β-catenin and alkaline phosphatase (Bae et al., 2013). The differentiation level of this phenotype is more advanced compared to the pre-odontoblasts originating from the dental papilla which only express β-catenin. Differential regulation of odontogenic differentiation could be the result of a dynamic interplay between Nfic and TGF-β1 which is highly dependent on the expression levels of both factors (He et al., 2014). TGF-β1 signaling promotes odontoblast differentiation via stimulation of the BMP/SMAD pathway and expression of DSPP (Iwamoto et al., 2017). On the other hand, TGF-β1 inhibits Nfic expression directly via SMAD3 upregulation and activation of the mitogen-activated protein kinase pathway which initiates Nfic degradation by SMURF1 and 2 (Lee et al., 2011). Nfic itself can counteract the inhibitory effect of TGF-β1 signaling by dephosphorylating the SMAD2/3 pathway. Comparing the expression levels of both signaling pathways during the progression of root elongation will shed more light on their close interaction.

## Heterogeneity in SCAP Phenotype—Lessons and Pitfalls

A vast amount of differentiation protocols have been successfully optimized in primary cell cultures of SCAP to demonstrate the high capacity of SCAP for osteogenic and adipogenic differentiation. SCAP are easily isolated from the apical papilla by tissue explant culture or by enzymatic digestion as demonstrated previously for dental pulp stem cells (Hilkens et al., 2013). However, to study the contribution of SCAP to dental root development, one should be cautious in interpreting the function of SCAP in culture. In tissue, SCAP are part of a micro-environmental niche which determines cell cycle, functional behavior and differentiation capacity (Diao et al., 2017). Orchestration of progressive root maturation is coordinated in conjunction with changes in the extracellular matrix and expression of growth factors (Huang et al., 2020). Gene expression analysis has proven that disruption of the micro-environmental niche leads to high number of differentially expressed genes when compared between tissue and SCAP in culture (Diao et al., 2017). SCAP from early passage have been shown to retain a more original phenotype as demonstrated by their intact mesodermal differentiation capacity (Rémy et al., 2019). A similar observation was made when studying Wnt inhibitory factor 1 (WIF1) expression, a Wnt/β-catenin modulator which maintains stem cell commitment (Wang and Cao, 2019). High expression levels of WIF1 are encountered within the native tissue of the apical papilla but decrease rapidly in primary cultures of SCAP. When overexpressing WIF1 in SCAP, it became apparent that the Wnt pathway promotes dentinogenic differentiation as indicated by the increased expression of odontogenic genes i.e., DSPP, DMP1, Runx2, and Osx (Wang and Cao, 2019). Prevention of cell culture induced adaptations in SCAP can be limited through either recreating tissue atmospheric conditions by lowering the oxygen concentration (Rémy et al., 2019) or by re-integration in a 3-D scaffold system (Somoza et al., 2017).

SCAP are identified as mesenchymal stem cells based on the expression of a standard panel of markers including Stro-1, CD24, CD29, CD73, CD90, CD105, CD106, CD146, ALP, and absence of expression of CD34, CD45, CD18, and CD150. Recent studies have underscored the presence of a heterogeneous pool of SCAP phenotypes residing in the apical papilla. When evaluating these phenotypes throughout literature, a distinction can be made based on the localization within the tissue. The majority of cells within the apical papilla express CD90 and a high concentration of CD105 and CD73 positive cells is located near the blood vessels (Ruparel et al., 2013). A further characterization within the CD146^+^ stem cell pool can be made based on STRO-1 expression (Bakopoulou et al., 2013). STRO1^+^ SCAP were shown to retain a high expression of embryonic and mesenchymal stem cell markers and enhanced odontogenic differentiation via activation of SMAD and Erk signaling. These characteristics were completely absent in the STRO1^–^ subpopulation. Importantly, the level of STRO-1 expression decreases progressively during increase in cell passages masking the original phenotype from the tissue. Treatment of SCAP using basic Fibroblast-Growth Factor (bFGF) maintains STRO-1 expression up to 10 passages, increases their proliferation, and preserves the undifferentiated state of the isolated stem cells (Wu et al., 2012). This process is regulated through binding of bFGF to FGF-receptors 1 or 2 expressed in SCAP (Chang et al., 2020) which initiate upregulation of oct4, Nanog and SOX2. These genes are highly involved in maintaining stem cell pluripotency and are part of a common stemness gene expression program observed in different types of dental stem cells originating from the bud and pulp tissue (Ballini et al., 2019). Furthermore, activation of FGF receptors enhances cell proliferation but negatively regulates mineralization by inhibiting Osx, osteocalcin, and ALP (Wu et al., 2012). Immunohistochemistry revealed the location of NOTCH3^+^ and CD146^+^ stem cells in the vicinity of the blood vessel walls (Jamal et al., 2015). The role of NOTCH3 has also been associated with the preservation of the undifferentiated state of stem cells and its expression is markedly present during tooth development (Zhang et al., 2008; Sun et al., 2014). FACS analysis identified 4 different SCAP subpopulations based on NOTCH3 and STRO-1 expression underscoring the heterogeneity of SCAP phenotypes in tissue (Jamal et al., 2015). Furthermore, CXCR4 is highly expressed in perivascular SCAP and is activated via SDF-1, indicating chemo-attraction (Liu J. Y. et al., 2015). SDF-1 activates phosphatidylinositol 3-kinase and protein kinase C signaling pathways in SCAP and contributes to the transmigration capacity (Chen et al., 2016). The latter is reflected by the formation of focal adhesions and the expression of focal adhesion molecules, i.e., p-focal adhesion kinase, p-paxillin, and vinculin, linking the adhesion complexes to a stress fiber network (Chen et al., 2016). In addition, the SDF-1/CXCR4 pathway contributes to induction of odontogenic differentiation via BMP-2 (Xiao et al., 2019).

## Interaction of SCAP With the Micro-Environment

From the aforementioned studies we can conclude that SCAP are a heterogeneous stem cell pool which have the capacity to migrate to the growing dental root and to differentiate into odontoblasts. However, the impact of SCAP is not only confined to mere differentiation but they also affect their surrounding micro-environment by high secretion of a plethora of factors. Analysis of the SCAP secretome in conditioned medium revealed a diverse collection of proteins consisting of chemokines along with angiogenic, immunomodulatory, anti-apoptotic, neuroprotective factors, and extracellular matrix (ECM) proteins (Yu et al., 2020). Moreover, SCAP produce components of the renin-angiotensin system (RAS) (Macedo et al., 2021) which could increase SCAP proliferation rate via angiotensin 2 receptor activation (Pizzatto et al., 2020). Several studies indicate the capacity of the apical papilla to maintain cells in a non-differentiated state intrinsically and even in neighboring tissues via currently unknown secretory signaling molecules. Our group discovered that the central peripheral nerve within the apical papilla is surrounded by a resident vimentin negative cell population in contrast to the abundant vimentin positive SCAP in the medulla (Driesen et al., 2020). Therefore, it is hypothesized that the vimentin negative cell fraction maintains the opening of the root canal preventing odontoblast differentiation and dentin deposition. Indeed, in a co-culture system, SCAP were able to negatively regulate osteogenic differentiation of dental follicle stem cells (Wu et al., 2018). The apical papilla would thus exert an inhibitory effect on dental follicle stem cells during the formation of the dental root.

Since progression of dental root growth is associated with a reduction in apical papilla size and eventually disappears after final root maturation (Figure 1), one can hypothesize that the extracellular matrix is prone to continuous remodeling and degradation. Thus, an intrinsic balance between collagen digestion and cross-linking could serve as a mechanism for SCAP migration and recruitment to the growing root. Collagen digestion requires the secretion of enzymes with a collagenase activity such as membrane-type matrix metalloproteinase 1 (MT1-MMP) which is a zinc-endopeptidase and exclusively expressed during dental formation within the dental mesenchyme (Xu et al., 2016). Reduced activity of MT1-MMP has led to impaired root formation and as a result incomplete tooth eruption. In addition, we identified abundant expression of fibroblast-activation protein-α in dental mesenchymal stem cells and in particular within SCAP (Driesen et al., 2020). FAP-α is a member of the family of cell surface serine proteases and is highly expressed during embryonic development and contributes to extracellular matrix remodeling via collagenase type I activity. The presence of FAP-α in SCAP could point to a capacity to re-organize their collagen-based micro-environment creating a suitable substrate for enhanced migration toward the developing root.

## Future Perspectives and Conclusion

Studies involved in comprehending the molecular mechanism of root formation has already provided novel insights in dental disorders. The pathology of short root anomaly is recognized by the development of abnormal short roots with a blunt appearance (Yu et al., 2021) and is a consequence of a dysregulation of nuclear factor 1 C-type, Osx, bone morphogenetic protein, TGF-β, Wnt-β catenin, and DKK1. Pulp necrosis and apical periodontitis in immature teeth negatively affects proper root development posing difficulties for proper dental treatment. Strategies are currently being investigated to restore or regenerate defective root formation. Berberine has been shown to induce root repair via activation of the Wnt/β-catenin pathway in SCAP leading to longer roots and thicker root walls (Cui et al., 2020). Mixture of SCAP and PDLSCs has proven to initiate regeneration of the root and periodontal structure in swine (Sonoyama et al., 2006). An interesting novel approach is the use of biomimetic scaffolds with flexible modeling of the scaffold’s geometry. Integration of SCAP into these scaffolds with a stem-cell matching microarchitecture could procure new therapeutic strategies for bio-artificial root replacement (Ballini et al., 2017). However, many processes in dental root maturation are not resolved at the moment. Studying the interaction between the apical papilla, HERS and dental follicle will shed more light on the micro-environmental changes and extracellular matrix remodeling associated with apical papilla remodeling during root maturation. Identification and mechanistic understanding of the cellular heterogeneity within the apical papilla will be an important first step toward the development of tissue engineered apical papillae for future dental root regeneration therapy.

## Author Contributions

RD conceived and wrote the manuscript. TV, PG, and IL reviewed the manuscript. All authors contributed to the article and approved the submitted version.

## Conflict of Interest

The authors declare that the research was conducted in the absence of any commercial or financial relationships that could be construed as a potential conflict of interest.
